# *In vitro* antibacterial effect of wasp (*Vespa orientalis*) venom

**DOI:** 10.1186/1678-9199-20-22

**Published:** 2014-05-20

**Authors:** Jafar Jalaei, Mehdi Fazeli, Hamid Rajaian, Seyed Shahram Shekarforoush

**Affiliations:** 1 Department of Pharmacology and Toxicology, School of Veterinary Medicine, Shiraz University, Shiraz, Iran; 2 Department of Food Hygiene, School of Veterinary Medicine, Shiraz University, Shiraz, Iran

**Keywords:** Wasp venom, *Vespa orientalis*, Antimicrobial activity, Minimum inhibitory concentration

## Abstract

**Background:**

The emergence of antibacterial resistance against several classes of antibiotics is an inevitable consequence of drug overuse. As antimicrobial resistance spreads throughout the globe, new substances will always be necessary to fight against multidrug-resistant microorganisms. Venoms of many animals have recently gained attention in the search for new antimicrobials to treat infectious diseases. Thefore, the present study aimed to study the antibacterial effects of wasp (*Vespa orientalis*) crude venom.

Two gram-positive bacteria (*Staphylococcus aureus* and *Bacillus subtilis*) and two gram-negative ones (*Escherichia coli* and *Klesiella pneumonia*) were compared for their sensitivity to the venom by determining the inhibition zone (Kirby-Bauer method) and minimum inhibitory concentration (MIC). A microbroth kinetic system based on continuous monitoring of changes in the optical density of bacterial growth was also used for determination of antimicrobial activity.

**Results:**

The venom exhibited a well-recognized antimicrobial property against the tested bacterial strains. The inhibition zones were determined to be 12.6, 22.7, 22.4 and 10.2 mm for *S. aureus*, *B. subtilis*, *E. coli* and *K. pneumonia*, respectively. The corresponding MIC values were determined to be 64, 8, 64 and 128 μg/mL, respectively. The MIC_50_ and MIC_90_ values of the venom were respectively determined to be 63.6 and 107 μg/mL for *S. aureus*, 4.3 and 7.0 μg/mL for *B. subtilis*, 45.3 and 65.7 μg/mL for *E. coli* and 74.4 and 119.2 μg/mL for *K. pneumonia.* Gram-positive bacteria were generally more sensitive to the venom than gram-negative ones.

**Conclusions:**

Results revealed that the venom markedly inhibits the growth of both gram-positive and gram-negative bacteria and could be considered a potential source for developing new antibacterial drugs.

## Background

Since the discovery of penicillin, numerous antibiotics have been developed, primarily to treat bacterial or fungal infections. This fact constituted an important contribution to human and animal health in the fight against infectious diseases [1,2]. The widespread and/or inappropriate use of antibiotics and chemicals against harmful microorganisms has led to microbial resistance [3]. The emergence of antibiotic-resistant organisms comprises a serious worldwide problem. Recent findings on new antibiotic-resistant organisms include multiple-drug resistant (MDR) *Pseudomonas aeruginosa*, MDR *Acinetobacter baumannii* and New Delhi Metallo-β-Lactamase-1 (NDM-1) producing bacteria. Kumarasamy *et al.*[4] reported that the ‘superbugs’ that produce NDM-1 were resistant to almost all antibiotics, except for polymyxin and tigecycline.

The augmentation in antibiotic resistance is a major challenge in medicine since not many new antibiotics are being produced, and bacteria resistant to currently available drugs are increasing. Seeking novel antibacterial substances and antibiotic combination therapy are the strategic options to overcome the MDR organisms [5,6]. Such initiative has greatly driven the search for new antimicrobial agents with novel mechanisms of action that are broadly effective and less likely to induce antimicrobial resistance. These drugs will be very important, particularly for the treatment of immune compromised patients [7-9].

Despite tremendous advances in biological sciences, the difficulty in identifying new mechanisms to kill bacterial pathogens is discouraging. Thus, finding alternative sources of new drugs or prototypes is of major interest to complementary medicine. In the hope of inventing novel antimicrobial agents to control antibiotic-resistant bacteria, natural products are an important source of medicinal compounds. A wide variety of organisms produces such bioactive compounds and some of these natural substances have been shown to be able to kill bacteria [10,11]. Venoms of a vast number of animal species represent complex mixtures of compounds (ions, biogenic amines, polyamines, polypeptide neurotoxins, cytolytic peptides, enzymes etc.) responsible for various effects [12-15]. Venoms can also be useful and valuable as pharmacological tools in drug research, as potential drug design templates and as therapeutic agents [16,17]. In recent years, venoms and venom components from animals have shown potential antibacterial activity. These include venom of wasps, common honeybees, spiders, snakes and scorpions [18-24]. Bearing in mind all these facts, the present study was conducted to evaluate the antibacterial activity of *Vespa orientalis* venom against different strains of gram-positive and gram-negative bacteria.

## Methods

### Venom extraction

*Vespa orientalis* specimens were collected from Abarkooh, Yazd Province, Central Iran. Wasps were paralyzed at 4°C, their venom glands were dissected and immersed in liquid nitrogen. The glands were then crushed in a clean mortar using pestle and liquid nitrogen. Twenty milliliters of 0.1 M buffer phosphate (pH = 7.4) was added to the powdered sample immediately after the evaporation of liquid nitrogen. The suspension was then transferred into a clean tube and further homogenized. Each tube containing the sample was centrifuged at 8,000 × *g* for 15 minutes at 4°C. The supernatant was transferred into another tube, lyophilized and kept at -20°C until further assay.

### Antimicrobial assay

#### Bacterial strains

The microorganisms used in the antibacterial screening assays were: gram-positive bacteria including *Staphylococcus aureus* ATCC 6538 and *Bacillus subtilis* ATCC 1010649, and gram-negative bacteria including *Escherichia coli* ATCC 35218 and *Klebsiella pneumonia* ATCC 700603. The bacteria were resuspended in tryptic soy broth (TSB), incubated at 37°C overnight and stored at 4°C*.*

#### Agar disc diffusion method

The screening of antimicrobial activity of the crude venom was carried out by agar disc diffusion method using Mueler-Hinton agar (Merck, Germany). Similarly, tetracycline was used for comparison. The bacterial inocula were prepared from the colonies of 24 hour-cultured bacteria on nutrient agar. The inocula were adjusted with McFarland density to obtain a final concentration of approximately 10^5^ CFU/mL. The Millipore filter paper discs (Millipore Corporation, USA) were impregnated with either crude venom or tetracycline (30 μg) and applied on the test media previously inoculated with each bacterial strain. Plates were incubated at 37°C and inhibition zones were measured after 24 hours of incubation.

#### Broth microdilution method

The minimum inhibitory concentration (MIC) of crude venom and tetracycline was also determined using conventional broth microdilution method according to the CLSI guidelines [25]. The adjusted bacterial suspensions were added to each well of sterile microtiter plate containing the test concentrations of antimicrobials (100 μL/well) in Mueler-Hinton broth (MHB, Merck, Germany). Consequently, final inoculum concentration of 1 × 10^5^ CFU/mL was obtained in each well and this plate was incubated for 24 hours at 37°C. The antimicrobial, a non-treated control, and a sterility control were also used. Each assay was carried out in triplicate. The lowest concentration of antibiotic which inhibited the visible bacterial growth was selected as MIC.

#### Growth curves

Antimicrobial activity of the crude venom was also examined using a 96-well sterile microtiter plate. The crude venom and tetracycline (Sigma, Germany) were serially diluted in MHB at concentrations of 1024, 512, 256, 128, 64, 32, 16, 8, 4 and 2 μg/mL and 16, 8, 4, 2, 1, 0.5, 0.25 and 0.125 μg/mL, respectively, in a final volume of 100 μL. Each well was inoculated with 10 μL of the bacterial suspension containing 10^6^ CFU/mL. Each test was performed in triplicate. Three wells containing bacterial suspension with no drug (growth control) and three wells containing high concentration of tetracycline (background control) were also included. Optical densities were measured for 24 hours at 37°C using a multidetection microplate reader (BioTek's PowerWave XS2, USA) at 600 nm and recorded automatically for each well every two hours. Turbidimetric growth curves were obtained depending on the changes in the optical density of bacterial growth for each drug concentration and the drug-free growth control.

For the determination of MIC of crude venom and tetracycline by the microbroth kinetic assay, the percentage of growth at each drug concentration was calculated using the following equation:

$$\%\;\mathrm{Inhibition}=\;100-\left[\frac{\text{OD}\;\text{of}\;\text{venom}\;\text{containing}\;\text{wells}‒\text{OD}\;\text{of}\;\text{background}\;\text{control}}{\text{OD}\;\text{of}\;\text{the}\;\text{growth}\;\text{control}‒\text{OD}\;\text{of}\;\text{background}\;\text{control}}\right]\times 100\;$$

## Results

**Figure 1:**
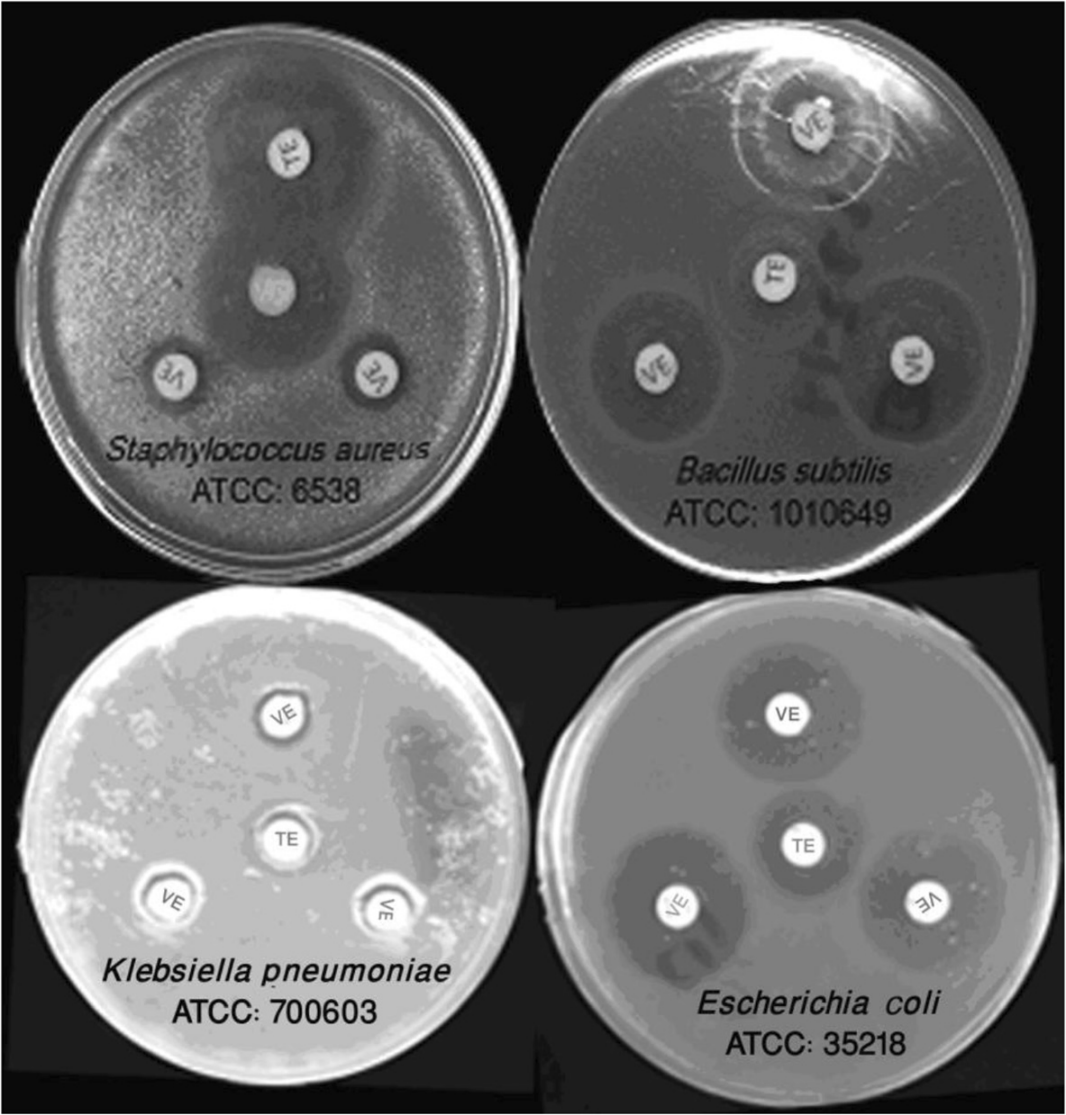
**Inhibitory effect of *Vespa orientalis *** **(VE: venom extract, 30 μg/disc) on the growth of different bacterial strains compared with tetracycline (TE: tetracycline, 30 μg/disc).**

**Figure 2:**
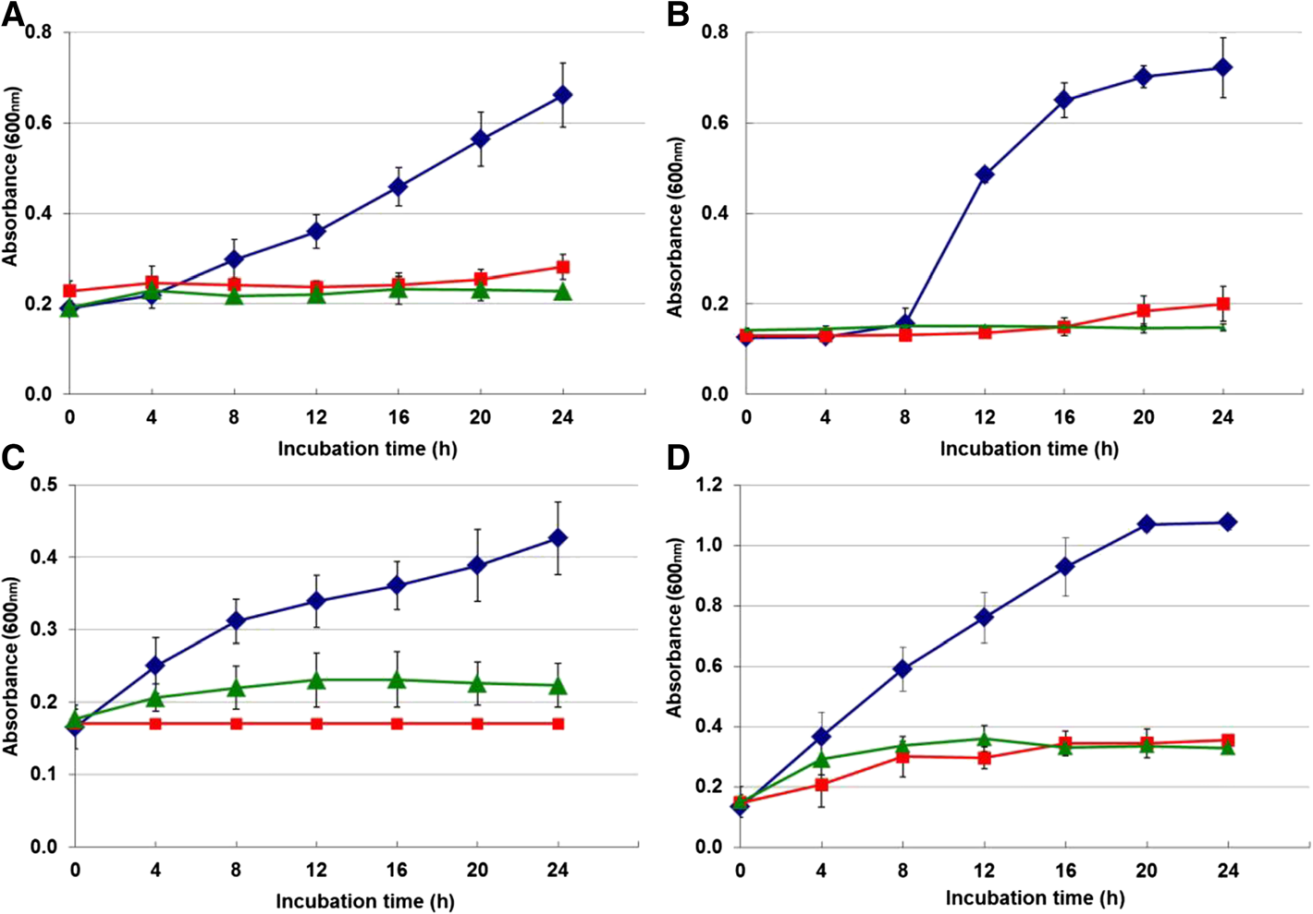
**Growth curves of different bacterial strains exposed to *Vespa orientalis *crude venom during 24 hours. (A) ***Staphylococcus aureus* with 64 μg/mL*, ***(B) ***Bacillus subtilis* with 8 μg/mL, **(C) ***Escherichia coli* with 64 μg/mL and **(D) ***Klesiella pneumonia* with 128 μg/mL compared with tetracycline with 16 μg/mL at 37°C. (blue diamond symbol): Control, (red square symbol): tetracyclin, (green triangle symbol): wasp crude venom.

**Figure 3:**
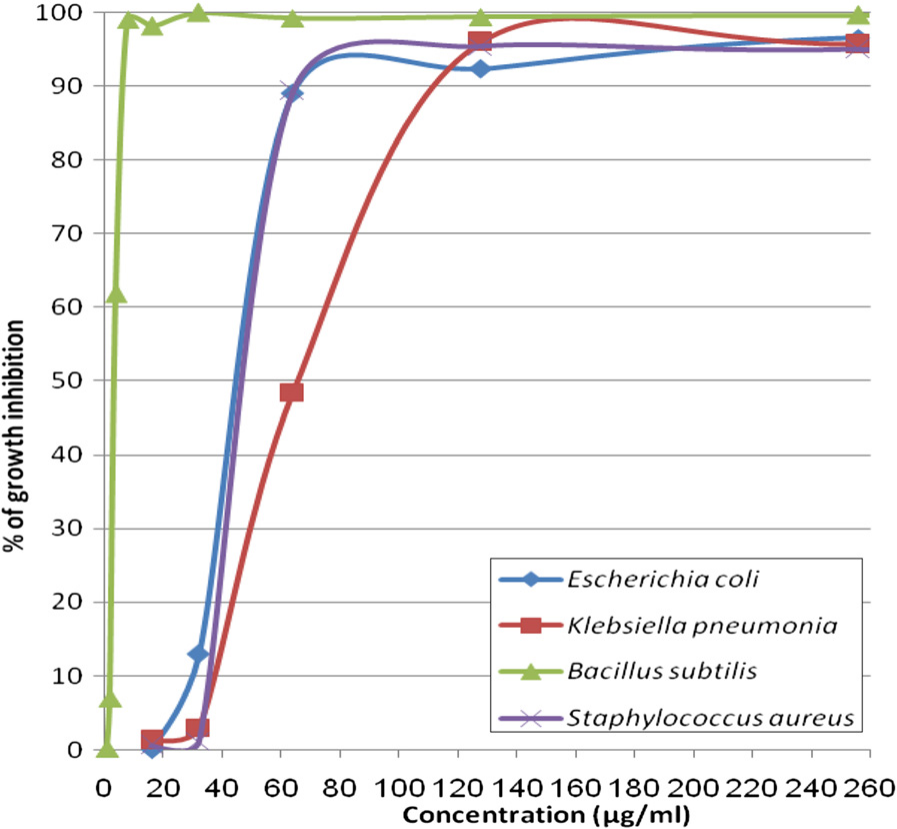
**Inhibitory effect of various concentrations of *Vespa orientalis *** **crude venom on the growth of different bacterial strains.**

*Vespa orientalis* crude venom displayed a significant effect against different gram-positive and gram-negative bacterial strains emplyed in this study. The corresponding inhibition zones and MICs are listed in Figure 1 and Table 1. The crude venom caused a marked inhibition in bacterial growth with inhibition zones of 12.6, 22.7, 22.4 and 10.2 mm for *S. aureus*, *B. subtilis*, *E. coli* and *K. pneumonia* respectively. The corresponding MICs of the crude venom were respectively found to be 64, 8, 64 and 128 μg/mL using the conventional microdilution method (Figure 1 and Table 1). Growth curves of different bacteria during the incubation period in the presence of various concentrations of crude venom are presented in Figure 1. The MIC_50_ and MIC_90_ of the crude venom against different bacteria determined by microbroth kinetic system were respectively 63.6 and 107 μg/mL for *S. aureus*; 4.3 and 7 μg/mL for *B. subtilis*; 45.3 and 65.7 μg/mL for *E. coli*; 74.4 and 119.2 μg/mL for *K. pneumonia* (Figures 2 and 3; Table 2). All tested bacterial strains were found to be susceptible to the venom and among them, *B. subtilis* was the most sensitive. In addition, the present findings indicate that the crude venom is more effective against gram-positive than gram-negative bacteria.

**Table 1:** **Inhibitory effect of *Vespa orientalis *** **crude venom on different strains of bacteria**

**Microorganisms**	**Inhibition zone**^**1 **^**(mm)**		**MIC**^**2 **^**(μL/mL)**	
	**Venom**	**Tetracycline**	**Venom**	**Tetracycline**
*S. aureus*	12.6 ± 0.31^3^	28.1 ± 0.64	64	0.125
*B. subtilis*	22.7 ± 0.62	18.9 ± 0.14	8	0.125
*E. coli*	22.4 ± 0.68	19.3 ± 1.75	64	0.5
*K. pneumonia*	10.2 ± 0.12	12.1 ± 0.13	128	1

^1^Growth inhibition effects of venom and tetracycline was determined by disc diffusion method (30 μg/disc). ^2^Minimum inhibitory concentrations were obtained using serial dilutions. ^3^Mean ± SD (n = 3).

**Table 2:** **Percent of inhibition action of different concentrations (μg/mL) of *Vespa orientalis *** **crude venom on the growth of different bacterial strains**

**Microorganisms**	**% inhibition**						
	**10**	**20**	**40**	**50**	**60**	**80**	**90**
*S. aureus*	20.2	31.1	52.8	63.6	74.5	96.2	107.0
*B. subtilis*	1.6	2.3	3.6	4.3	5.0	6.3	7.0
*E. coli*	24.9	30.0	40.2	45.3	50.4	60.6	65.7
*K. pneumonia*	29.6	40.8	63.2	74.4	85.6	108.0	119.2

## Discussion

The present study describes the assessment of antimicrobial effects of *Vespa orientalis* crude venom using the microbroth kinetic system, which determines the effect of antibacterial agents by continuous monitoring of bacterial culture optical density. The crude venom exhibited activity against both gram-positive and gram-negative bacteria and the MICs obtained in microbroth kinetic system were similar to those found using conventional broth microdilution method. A shorter incubation period was required in the former technique and percentages of growth inhibition were also measurable.

To address the rapid emergence of resistance to the classical antibiotics, naturally occurring antibacterial agents are promising candidates in the search for novel therapeutic agents [26]. Antibacterial property has been reported for the venoms of a wide variety of animals including venoms of snakes, scorpions, spiders, conus and wasps all of which are predatory or parasitic animals [27-30]. However, the actual function of antimicrobial agents in these venoms is not clear yet.

Venom components from predator wasps including hornets (genera *Vespa* and *Dolichovespula*), yellow jackets (genus *Vespula*) and paper wasps (genus *Polistes*) have been extensively studied. Their toxins are complex mixtures of amines, small peptides and high molecular weight proteins such as enzymes, allergens and toxins [31-33]. Venoms from these stinging wasps are important weapons both in the defense of the colony and capture of prey. To the best of our knowledge, only a few of its components have been purified and characterized from parasitic Hymenoptera, such as metalloproteinase, serpin, calreticulin-like protein, aspartyl glucosaminidase-like protein and insecticidal toxins [34-38].

The antimicrobial property of wasp venoms is mostly due to their peptides. Amphipathic secondary structures with net positive charges are essential to the biological activities of peptides that interact with anionic components of bacterial membranes in different ways, sometimes resulting in irreversible damage to the cell [39].

One of the major targets for antimicrobial agents is the bacterial cell envelope, which is a complex, multiple macromolecular structures that undergoes highly ordered cycles of synthesis and hydrolysis, facilitating cell division while maintaining a protective barrier against environmental stress. There are several different classes of antibiotics that target specific cell envelope structures or enzymatic steps of cell wall synthesis [40]. The biological membrane is a highly dynamic, complicated system, which is composed of weakly interacting protein molecules and lipids [41].

Results of the present study revealed that wasp venom is more effective against gram-positive than gram-negative bacteria, which might be related to the difference in cell envelope structure. Cell wall of bacteria comprises a complex structure that is fundamentally different between gram-positive and gram-negative bacteria. It consists of a polymer of disaccharides cross-linked by short chain peptides, forming a type of peptidoglycan. Cell wall in gram-positive bacteria is thick (15–80 nm), consisting of several layers of peptidoglycans and molecules of teichoic acids. In contrast, cell wall of gram-negative bacteria is relatively thin (10 nm) in and is composed of a single layer of peptidoglycan surrounded by a membranous structure (the outer membrane) which may invariably contain lipopolysaccharides. Thus, the outer membrane is more hydrophobic in gram-negative than in gram-positive bacteria and constitutes a target for being attacked by hydrophobic agents and other antibiotic agents [42,43].

## Conclusions

*Vespa orientalis* crude venom efficiently inhibited the growth of gram-positive and gram-negative bacterial strains, even at a very low concentration when compared to that of tetracycline. The crude venom showed to be more efficient against gram-positive bacteria. As the crude venom is comprised of different proteins and peptides, further investigation is required to determine the potential components that could be used as antimicrobial drugs, especially for treating antibiotic-resistant pathogens.

### Ethics committee approval

The present study was approved by the Ethics Committee for Animal Experiments of Shiraz University.

## Competing interests

The authors declare that there are no competing interests.

## Authors’ contributions

JJ, PhD student of pharmacology, Shiraz University, collected the wasps and carried out most pharmacological and biochemical experiments; MF and HR designed the study, supervised the conduction of all experiments, drafted the manuscript and discussed the results. SSS helped conducting some experiments and performed statistical analyses. All authors read and approved the manuscript.

## References

[B1] Gordon YJ Romanowski EG McDermott AM A review of antimicrobial peptides and their therapeutic potential as anti-infective drugs Curr Eye Res 2005 30 7 505 515 10.1080/02713680590968637 16020284 PMC1497874

[B2] Wright GD The antibiotic resistome: the nexus of chemical and genetic diversity Nat Rev Microbiol 2007 5 175 186 10.1038/nrmicro1614 17277795

[B3] Fleet GH Spencer JFT, Spencer DM Food spoilage yeasts Yeast Technology 1990 Berlin: Springer

[B4] Kumarasamy KK Toleman MA Walsh TR Bagaria J Butt F Balakrishnan R Chaudhary U Doumith M Giske CG Irfan S Krishnan P Kumar AV Maharjan S Mushtaq S Noorie T Paterson DL Pearson A Perry C Pike R Rao B Ray U Sarma JB Sharma M Sheridan E Thirunarayan MA Turton J Upadhyay S Warner M Welfare W Livermore DM Emergence of a new antibiotic resistance mechanism in India, Pakistan, and the UK: a molecular, biological, and epidemiological study Lancet Infect Dis 2010 10 9 597 602 10.1016/S1473-3099(10)70143-2 20705517 PMC2933358

[B5] Anderson ET Young LS Hewitt WL Antimicrobial synergism in the therapy of gram-negative rod bacteremia Chemotherapy 1978 24 1 45 54 10.1159/000237759 412648

[B6] Kreger BE Craven DE McCabe WR Gram-negative bacteremia. IV. Re-evaluation of clinical features and treatment in 612 patients Am J Med 1980 68 3 344 355 10.1016/0002-9343(80)90102-3 6987871

[B7] Guardabassi L Kruse H Overlooked aspects concerning development and spread of antimicrobial resistance. Central European symposium on antimicrobial resistance, Brijuni, Croatia, 4–7 July, 2003 Expert Rev Anti Infect Ther 2003 1 3 359 362 10.1586/14787210.1.3.359 15482133

[B8] Hancock RE Diamond G The role of cationic antimicrobial peptides in innate host defences Trends Microbiol 2000 8 9 402 410 10.1016/S0966-842X(00)01823-0 10989307

[B9] Hujer AM Bethel CR Hujer KM Bonomo RA Antibiotic resistance in the institutionalized elderly Clin Lab Med 2004 24 2 343 361 10.1016/j.cll.2004.03.005 15177844

[B10] Wenhua R Shuangquan Z Daxiang S Kaiya Z Guang Y Induction, purification and characterization of an antibacterial peptide scolopendrin I from the venom of centipede *Scolopendra subspinipes multilans* Indian J Biochem Biophys 2006 43 88 93 16955756

[B11] Perumal Samy R Pachiappan A Gopalakrishnakone P Thwin MM Hian YE Chow VT Bow H Weng JT *In vitro* antibacterial activity of natural toxins and animal venoms tested against *Burkholderia pseudomallei* BMC Infect Dis 2006 6 1 16 10.1186/1471-2334-6-1 16784542 PMC1569838

[B12] Corzo G Villegas E Gómez-Lagunas F Possani LD Belokoneva OS Nakajima T Oxyopinins, large amphipathic peptides isolated from the venom of the wolf spider *Oxyopes kitabensis* with cytolytic properties and positive insecticidal cooperativity with spider neurotoxins J Biol Chem 2002 277 26 23627 23637 10.1074/jbc.M200511200 11976325

[B13] Adams ME Herold EE Venema VJ Two classes of channel-specific toxin from funnel web spider venom J Comp Physiol A 1989 164 3 333 342 10.1007/BF00612993 2709340

[B14] Chan TK Geren CR Howell DE Odell GV Adenosine triphosphate in tarantula spider venoms and its synergistic effect with the venom toxin Toxicon 1975 13 1 61 66 10.1016/0041-0101(75)90159-2 1052562

[B15] Wullschleger B Nentwig W Kuhn-Nentwig L Spider venom: enhancement of venom efficacy mediated by different synergistic strategies in *Cupiennius salei* J Exp Biol 2005 208 Pt 11 2115 2121 15914655 10.1242/jeb.01594

[B16] Harvey AL Robertson B Dendrotoxins: structure-activity relationships and effects on potassium ion channels Curr Med Chem 2004 11 23 3065 3072 10.2174/0929867043363820 15579000

[B17] Koh DC Armugam A Jeyaseelan K Snake venom components and their applications in biomedicine Cell Mol Life Sci 2006 63 24 3030 3041 10.1007/s00018-006-6315-0 17103111 PMC11135979

[B18] Dani MP Richards EH Isaac RE Edwards JP Antibacterial and proteolytic activity in venom from the endoparasitic wasp *Pimpla hypochondriaca* (Hymenoptera: Ichneumonidae) J Insect Physiol 2003 49 10 945 954 10.1016/S0022-1910(03)00163-X 14511827

[B19] Perumal SR Gopalakrishnakone P Thwin MM Chow TK Bow H Yap EH Thong TW Antibacterial activity of snake, scorpion and bee venoms: a comparison with purified venom phospholipase A2 enzymes J Appl Microbiol 2007 102 3 650 659 10.1111/j.1365-2672.2006.03161.x 17309613

[B20] Fennell JF Shipman WH Cole LJ Antibacterial action of a bee venom fraction (melittin) against a penicillin-resistant *Staphylococcus* and other microorganisms. USNRDL-TR-67-101 Res Dev Tech Rep 1967 5 1 13 5300771 10.21236/ad0658324

[B21] Benli M Yigit N Antibacterial activity of venom from funnel web spider *Agelena labyrinthica* (Araneae: Agelenidae) J Venom Anim Toxins incl Trop Dis 2008 17 4 641 650 Available at: http://www.scielo.br/scielo.php?script=sci_arttext&pid=S1678-91992008000400007

[B22] Budnik BA Olsen JV Egorov TA Anisimova VE Galkina TG Musolyamov AK Grishin EV Zubarev RA De novo sequencing of antimicrobial peptides isolated from the venom glands of the wolf spider *Lycosa singoriensis* J Mass Spectrom 2004 39 2 193 201 10.1002/jms.577 14991689

[B23] Stiles BG Sexton FW Weinstein SA Antibacterial effects of different snake venoms: purification and characterization of antibacterial proteins from *Pseudechis australis* (Australian king brown or mulga snake) venom Toxicon 1991 29 9 1129 1141 10.1016/0041-0101(91)90210-I 1796476

[B24] Torres-Larios A Gurrola GB Zamudio FZ Possani LD Hadrurin, a new antimicrobial peptide from the venom of the scorpion *Hadrurus aztecus* Eur J Biochem 2002 267 16 5023 5031 10931184 10.1046/j.1432-1327.2000.01556.x

[B25] Wikler MA Cockerill FR Craig WA Dudley MN Eliopoulos GM Hecht DA Hindler JF Ferraro JM Swenson JM Low DE Sheehan DJ Tenover FC Turnidge JD Weinstein MP Zimmer BL Methods for dilution antimicrobial susceptibility tests for bacteria that grow aerobically, approved standard, Volume 26 2006 7 Wayne (PA): Clinical and Laboratory Standards Institute

[B26] Zasloff M Antimicrobial peptides of multicellular organisms Nature 2002 415 6870 389 395 10.1038/415389a 11807545

[B27] Xu C Ma D Yu H Li Z Liang J Lin G Zhang Y Lai R A bactericidal homodimeric phospholipases A2 from *Bungarus fasciatus* venom Peptides 2007 28 5 969 973 10.1016/j.peptides.2007.02.008 17383773

[B28] Gao B Xu J Rodriguez Mdel C Lanz-Mendoza H Hernández-Rivas R Du W Zhu S Characterization of two linear cationic antimalarial peptides in the scorpion *Mesobuthus eupeus* Biochimie 2010 92 4 350 359 10.1016/j.biochi.2010.01.011 20097251

[B29] Yan L Adams ME Lycotoxins, antimicrobial peptides from venom of the wolf spider *Lycosa carolinensis* J Biol Chem 1998 273 4 2059 2066 10.1074/jbc.273.4.2059 9442044

[B30] Biggs JS Rosenfeld Y Shai Y Olivera BM Conolysin-Mt: a conus peptide that disrupts cellular membranes Biochemistry 2007 46 44 12586 12593 10.1021/bi700775p 17927208

[B31] de Graaf DC Aerts M Danneels E Devreese B Bee, wasp and ant venomics pave the way for a component-resolved diagnosis of sting allergy J Proteomics 2009 72 2 145 154 10.1016/j.jprot.2009.01.017 19344653

[B32] Nakajima T Tu AT Handbook of Natural Toxins 1984 Volume 2 New York: Marcel Dekker

[B33] Habermann E Bee and wasp venoms Science 1972 177 4046 314 322 10.1126/science.177.4046.314 4113805

[B34] Price DR Bell HA Hinchliffe G Fitches E Weaver R Gatehouse JA A venom metalloproteinase from the parasitic wasp *Eulophus pennicornis* is toxic towards its host, tomato moth (*Lacanobia oleracae*) Insect Mol Biol 2009 18 2 195 202 10.1111/j.1365-2583.2009.00864.x 19320760

[B35] Colinet D Dubuffet A Cazes D Moreau S Drezen JM Poirié M A serpin from the parasitoid wasp *Leptopilina boulardi* targets the *Drosophila phenoloxidase* cascade Dev Comp Immunol 2009 33 5 681 689 10.1016/j.dci.2008.11.013 19109990

[B36] Zhang G Schmidt O Asgari S A calreticulin-like protein from endoparasitoid venom fluid is involved in host hemocyte inactivation Dev Comp Immunol 2006 30 9 756 764 10.1016/j.dci.2005.11.001 16364437

[B37] Moreau SJ Cherqui A Doury G Dubois F Fourdrain Y Sabatier L Bulet P Saarela J Prevost G Giordanengo P Identification of an aspartylglucosaminidase-like protein in the venom of the parasitic wasp *Asobara tabida* (Hymenoptera: Braconidae) Insect Biochem Mol Biol 2004 34 5 485 492 10.1016/j.ibmb.2004.03.001 15110870

[B38] Quistad GB Nguyen Q Bernasconi P Leisy DJ Purification and characterization of insecticidal toxins from venom glands of the parasitic wasp, Bracon hebetor Insect Biochem Mol Biol 1994 24 10 955 961 10.1016/0965-1748(94)90132-5 7703987

[B39] Sforça ML Oyama S Jr Canduri F Lorenzi CC Pertinhez TA Konno K Souza BM Palma MS Ruggiero-Neto J Azevedo WF Jr Spisni A How C-terminal carboxyamidation alters the biological activity of peptides from the venom of the eumenine solitary wasp Biochemistry 2004 43 19 5608 5617 10.1021/bi0360915 15134435

[B40] Jordan S Hutchings MI Mascher T Cell envelope stress response in Gram-positive bacteria FEMS Microbiol Rev 2008 32 1 107 146 10.1111/j.1574-6976.2007.00091.x 18173394

[B41] Esteban-Martin S Salgado J Self-assembling of peptide/membrane complexes by atomistic molecular dynamics simulations Biophys J 2007 92 3 903 912 10.1529/biophysj.106.093013 17085495 PMC1779969

[B42] Schwarz G Reiter R Negative cooperativity and aggregation in biphasic binding of mastoparan X peptide to membranes with acidic lipids Biophys Chem 2001 90 3 269 277 10.1016/S0301-4622(01)00149-1 11407644

[B43] Singleton P Bacteria in Biology, Biotechnology And Medicine 2004 Chichester: John Wiley & Sons

